# The Role of Botanical Families in Medicinal Ethnobotany: A Phylogenetic Perspective

**DOI:** 10.3390/plants10010163

**Published:** 2021-01-15

**Authors:** Airy Gras, Oriane Hidalgo, Ugo D’Ambrosio, Montse Parada, Teresa Garnatje, Joan Vallès

**Affiliations:** 1 Institut Botànic de Barcelona (IBB, CSIC-Ajuntament de Barcelona), Passeig del Migdia s.n., Parc de Montjuïc, 08038 Barcelona, Catalonia, Spain; oriane.hidalgo@csic.es (O.H.); tgarnatje@ibb.csic.es (T.G.); 2 Mediterranean Ethnobiology Programme Director, Global Diversity Foundation, 37 St. Margarets Street, Canterbury, Kent CT1 2TU, UK; ugotopia@yahoo.com; 3 Laboratori de Botànica (UB)—Unitat associada al CSIC, Facultat de Farmàcia i Ciències de l’Alimentació, Institut de Recerca de la Biodiversitat—IRBio, Universitat de Barcelona, Av. Joan XXIII 27-31, 08028 Barcelona, Catalonia, Spain; montse.parada@gmail.com; 4 Secció de Ciències Biològiques, Institut d’Estudis Catalans, Carrer del Carme 47, 08001 Barcelona, Catalonia, Spain

**Keywords:** botanical families, Catalan-speaking territories, database, diversity indices, ethnobotany, ethnomedicine, medicinal plants, phylogeny

## Abstract

Studies suggesting that medicinal plants are not chosen at random are becoming more common. The goal of this work is to shed light on the role of botanical families in ethnobotany, depicting in a molecular phylogenetic frame the relationships between families and medicinal uses of vascular plants in several Catalan-speaking territories. The simple quantitative analyses for ailments categories and the construction of families and disorders matrix were carried out in this study. A Bayesian approach was used to estimate the over- and underused families in the medicinal flora. Phylogenetically informed analyses were carried out to identify lineages in which there is an overrepresentation of families in a given category of use, i.e., hot nodes. The ethnobotanicity index, at a specific level, was calculated and also adapted to the family level. Two diversity indices to measure the richness of reported taxa within each family were calculated. A total of 47,630 use reports were analysed. These uses are grouped in 120 botanical families. The ethnobotanicity index for this area is 14.44% and the ethnobotanicity index at the family level is 68.21%. The most-reported families are Lamiaceae and Asteraceae and the most reported troubles are disorders of the digestive and nutritional system. Based on the meta-analytic results, indicating hot nodes of useful plants at the phylogenetic level, specific ethnopharmacological research may be suggested, including a phytochemical approach of particularly interesting taxa.

## 1. Introduction

Ethnobotany is a relatively recent denomination for a discipline that studies plant names, uses and management by human societies from ancient to current times, aiming at their projection to the future [1]. Even if a precedent of this term, botanical ethnography, was coined to name the investigation of any plant materials in archaeology in order to unveil their uses and symbolisms [2], Harshberger [1] himself emphasised the fact that ethnobotanical findings should not only constitute an inventory of old knowledge, but should be relevant for current productive activities. From those dates to the present times, ethnobotany undertook methodological innovations, but maintained the double approach of recording and preserving the ancient uses of plants by people—which contributes to describing human lifestyles—and aiming to improve human life conditions [3]. This is why the collection of plant uses related to health, mostly medicinal and food ones, are predominant in ethnobotanical research, although other uses are relevant as well [4]. According to the importance of using folk local knowledge to preserve and improve health, not a few drugs have been developed based on their ethnobotanical background, such as, to quote just two famous and recent ones, the oseltamivir, used against chicken flu [5], and the antimalarial artemisinin [6]. Additionally, in agreement with this, medical or pharmaceutical ethnobotany, the botanical side of ethnopharmacology, is one of the main pillars of the discipline, particularly in industrialised countries [7,8].

In Europe, the Catalan linguistic domain, the framework of the present research, is among the better-known Iberian areas from an ethnobotanical viewpoint [9]. The amount of information recollected until now allows us to start conducting research involving comparison among several territories [4,10], in order to establish general patterns in ethnobotanical knowledge. 

In the above-referred geographical area, as in general in Europe and worldwide, the predominant ethnobotanical research has been an ethnofloristic one [11,12]. Nevertheless, efforts are being devoted toward finding other complementary approaches, such as studies focused on plants used for ailments related to a determined system, and on the validation of the ethnobotanical evidence with chemical or pharmacological data [13]. Moreover, the potentially predictive role of molecular phylogeny in bioprospection and in phytochemistry [14,15] and the concept of ethnobotanical convergence [16,17] has opened the way to integrate ethnobotany with genetic (including molecular phylogenetic), genomic (and other “omic” disciplines) and phytochemical approaches [18,19]. 

One of the aspects always addressed in ethnobotanical investigation is the distribution of the plant taxa recorded in botanical families, since, after the number of plant species known in a given territory, the families in which they are included is one of the most evident pieces of information. Even if some deeper analyses of the causes for some predominating families have been undertaken [18,20,21,22,23], work in this field is still lacking, including territories other than those already considered, and taking into account, among others, phylogenetic issues. 

Several statistical methods have been used to test whether a specific taxonomic group is over- or underrepresented in an ethnobotanical flora in comparison to overall local flora. Although the linear regression analysis [21] and binomial analysis [24] have been widely used, more recent studies have pointed out some limitations in these previous analytical methods, and propose a Bayesian approach in order to analyse the over- and underused plant groups [22,23]. This method allows us to consider the uncertainty of the proportion of medicinal plant species in the overall flora and shows its robustness in small datasets [22].

In this context, the aim of this work is to shed light on the role of botanical families in ethnobotany, depicting, in a molecular phylogenetic frame, the relationships between botanical families and medicinal uses of vascular plants in several Catalan-speaking territories (Formentera, Mallorca, and Catalonia, Northern Catalonia included). This will allow us to ascertain the most used families in pharmaceutical ethnobotany in this area and the possible phylogenetic reasons accounting for this, and to find out whether some families are more focused on some particular health conditions than others. 

## 2. Results and Discussion

For the areas under consideration, we analysed a total of 47,630 use reports corresponding to medicinal uses of vascular plants in human medicine registered in our database, including data from ethnobotanical research performed from 1991 until today, in order to study different aspects related to the botanical families to which these uses belong, and the disorders they refer to.

Based on these data, we can state that the medical ethnobotany of the Catalan-speaking territories scrutinised is distributed in 120 botanical families of vascular plants (seven to pteridophytes, five to gymnosperms and 108 to angiosperms). These families include 894 taxa, with medicinal uses, of which 41 have only been determined at generic level (the remaining majority, at specific and infraspecific levels).

The ethnobotanicity index (EI) for the studied area is 14.44%. In addition, the total considered flora ([25], see materials and methods for precisions) is at least slightly larger than that of the territories object of the present research, since some plants present in the places not covered here (i.e., Valencian area and some of the Balearic Islands) do not grow in the areas here concerned. This just means that, in fact, the EI in the studied area has been slightly underestimated. The result, as calculated, is higher than in other Mediterranean areas like Arrábida Natural Park, Portugal (12.1%; [26]), Monti Sicani Regional Park, Italy (12.7%, [27]) or the northwest Basque Country (12%; [28]), and lower than that of Serra de São Mamede, Portugal (23.1%; [29]), in the same biogeographical region, or that of Keelakodankulam, India (20.17%; [30]), in a quite distant and floristically different area.

The EI was conceived [31], and is usually calculated, for species and infraspecific taxa. Nevertheless, as the present paper focuses on families too, we calculated the EI for this taxonomic category (excluding the 15 non-native families, not present in the flora used as a basis). We found a familiar EI of 68.21%, indicating that almost three-quarters of the families in the area considered contained plants used in pharmaceutical ethnobotany. We believe that it would be of interest to calculate this parameter for other ethnofloras, in order to be able to compare the rates of families hosting useful plants.

### 2.1. Most Reported Families

Among the 10 most cited families (Table 1), which represent 57.34% of total use reports, we find some of the most relevant in ethnobotanical studies in the Mediterranean, namely Lamiaceae (15.40%), Asteraceae (11.90%) or Rosaceae (5.57%). These are large in terms of number of taxa, Asteraceae being the largest one [32]. These families are cosmopolitan and well represented in our territories, but also, particularly in Lamiaceae and Rosaceae, they are economically very significant, thanks to aromatic plants in Lamiaceae [33] and edible fruits and ornamental uses in Rosaceae [34]. In ethnofloristic works conducted in Mediterranean areas, these three families are almost always predominate at the top of the list, and also some others such as Fabaceae and Apiaceae [11,12,26,35,36,37,38,39,40,41,42,43,44,45]. From this, the simple but clear idea can be deduced, that people use with preference (or at least importantly) the plants that they easily find not far from their place of daily life, as Johns et al. [46] and Bonet et al. [47] stated.

In addition, partly due to the restructuring of families following the APG IV last update [48], in which Sterculiaceae and Tiliaceae have become part of the family Malvaceae, this latter family also appears in the top 10 most reported families, with 4.82%. 

The remaining most quoted families are Adoxaceae (4.14%), Apiaceae (3.49%), Amaryllidaceae (3.24%), Oleaceae (3.08%), Pinaceae (2.90%) and Rutaceae (2.79%). Four of these families (Apiaceae, Oleaceae, Pinaceae, Rutaceae) have not been the object of any recent systematic restructuring, and they are classically important in terms of medicinal taxa. Conversely, the Adoxaceae, with only ca. 225 species worldwide [49], and just six of them in the studied area [25], now host *Sambucus nigra*, one of the most used plants in the Mediterranean region and, in particular, in the Catalan-speaking territories [50,51], which has recently been transferred from the Caprifoliaceae. Similarly, the Amaryllidaceae exhibit a significant rate owing to the fragmentation of the Liliaceae *lato sensu* in several families and the attribution of genus *Allium* to this family, which is also very relevant in Mediterranean pharmaceutical ethnobotany [42]. 

Moerman [20] concluded that although in a random universe the size of a family would be the best predictor of its medicinal potential in number of taxa, the Asteraceae contain more medicinal plants than random would indicate, so that size is not the only condition for this success. Moerman et al. [21] found, through a comparative analysis of several geographically distant medicinal floras, that the five most important medicinal plant families in four very differentiated regions (North America, Korea, Kashmir, Chiapas highlands) were delineated by only nine plant families (Araceae, Bignoniaceae, Ericaceae, Euphorbiaceae, Fabaceae, Loganiaceae, Malvaceae, Rosaceae, Solanaceae), accounting for the existence of a global pattern of human knowledge. Indeed, to include a fifth area (Ecuador), only three more families were necessary (Apiaceae, Asteraceae, Lamiaceae). In the same line, six out of the top ten families in the present study are among the 14 most quoted ones in the five North American, Mesoamerican, South American and Asian territories investigated by Moerman et al. [21], those reported above plus Liliaceae and Ranunculaceae. The only top families in this paper not appearing in Moerman et al. [21] are Adoxaceae, Oleaceae, Pinaceae and Rutaceae (considering the Amarillydaceae included in the Liliaceae *lato sensu*, as this family was referred to in the aforementioned work).

### 2.2. Over- and Underuse of Plant Groups and Plant Families

Results for the over- and underused high taxonomic groups are shown in Table 2. Gymnosperms and monocots are the only two groups that differ from the common proportion. The observed proportion was 0.1709 and ranges from 16% and 18% with 95% of probability and, consequently, we can refuse the null hypothesis for these groups. While monocots are underused, gymnosperms are overused. The proportion of used monocots is very low, and the 95% posterior credible interval very narrow. Contrarily, gymnosperms show the highest proportion of the used plants, and a larger interval, possibly related, according to Weckerle et al. [22], to their small number of species. 

The families whose 95% posterior credible interval lies above the interval of the overall proportion of flora (0.160, 0.182) are listed in Table 3. These overused families are families represented by a small number of genera, and most of them having medicinal uses, that is, with high proportions. The preponderance of woody species over herbaceous among the most used has been discussed by several authors [20,52,53,54]. Fagaceae, Rutaceae and Cannabaceae are the three most overused families. The woody families such as Fagaceae, Pinaceae and Cupressaceae (the last two gymnosperms) together with some shrubby families such as Buxaceae, Rhamnaceae and Ericaceae are overrepresented in the medicinal Catalan flora, but in approximately the same proportion as weedy plant families, such as Equisetaceae, Asphodelaceae or Urticaceae. 

The families whose 95% posterior credible interval lies below the interval of the overall proportion of flora (0.160, 0.182) are listed in Table 4. Usually, these are families comprising a large number of species in the local flora and with little representation in the medicinal one. In the present study, only nine families are underused, Cyperaceae, Plumbaginaceae and Poaceae, the most underused. Three families, Poaceae, Juncaceae and Cyperaceae belong to the underrepresented high group of monocots. In the present study, most of members of the underused families are herbaceous plants. 

### 2.3. Genera with Folk Medicinal Uses

The 120 families recorded are represented herein by 432 genera. If we analyse the number of genera per family, the results vary slightly, and families with more genera are Asteraceae (52), Fabaceae (30) and Lamiaceae (25). Apart from the two families with the most use reports, here appears the Fabaceae family (765 UR, 1.61%), although not being among the top ten. Despite not having a high percentage, Fabaceae is a relevant family in the Mediterranean flora (even being indicative of the Mediterranean character of a territory; [55]) and ethnoflora [42]. Conversely, Adoxaceae with a large number of use reports (1,971 UR, 4.14%) is very asymmetrically distributed in only two genera, *Sambucus* (99.9%) and *Viburnum* (0.1%), which would be explained by the change of family, since they previously belonged to the Caprifoliaceae, yet with the APG system, a new familiar delimitation of the Adoxaceae was created for these two genera, and for three more not present in our territory. On the other hand, there are 60 botanical families in which all the reports are grouped in a single genus. Some examples, representing pteridophytes, gymnosperms and angiosperms, are the Equisetaceae, a family with 578 UR exclusive of the genus *Equisetum*, the only one in the family to be present in the studied area, and the Taxaceae and the Juglandaceae families, with 17 and 570 UR respectively, exclusively represented by their corresponding single species growing in the area, *Taxus baccata* L. and *Juglans regia* L. There are also families that concentrate all the medicinal records in one genus, although they have other representatives in the territories studied like Liliaceae *stricto sensu,* with six genera in the concerned area, but with all UR from only one, *Lilium*.

### 2.4. Plants not Appearing in the Flora of the Studied Area

Concerning plants not appearing in the flora of the territories studied ([25]; see materials and methods for details), there are 15 families not present in the flora of our territory, representing 12.5% of the total number of families. Examples are Actinidiaceae, Myristicaceae and Zingiberaceae. These families contain very renowned and used medicinal plants, some of them used first for food purposes, such as *Actinidia chinensis* Planch., *Myristica fragrans* Houtt. and *Zingiber officinalis* Roscoe. In addition, there are 62 taxa not present in our territory, yet belonging to families that nonetheless are present thanks to other genera; this is the case of *Cinnamomum verum* J.Presl (Lauraceae), *Cocos nucifera* L. (Arecaceae) or *Coffea arabica* L. (Rubiaceae), to give some examples. Even the two most quoted families, in terms of UR, Asteraceae and Lamiaceae, very abundant in wild representatives in the region under investigation, include non-native plants in the local ethnoflora, some examples are *Echinacea purpurea* (L.) Moench and *Stevia rebaudiana* (Bertoni) Bertoni, and *Monarda didyma* L., *Ocimum basilicum* L., and *Perilla frutescens* (L.) Britton, respectively. It is important to emphasise that a number of plants ethnobotanically used are not present in the Catalan flora [25], and yet they are important in local ethnobotany. Indeed, in a work in progress regarding in the Catalan linguistic area we are recording a non-negligible number and percentage of UR attributed to non-native plants [56].

### 2.5. Most Reported Troubles

We grouped the troubles or systems addressed in 15 categories (Table 5). The four most addressed troubles, representing 61.01% of all use reports, are disorders of the digestive and nutritional system (11,754 UR, 24.68%), followed at a great distance (approximately half of the use reports) by respiratory system disorders (6418 UR, 13.47%), skin or subcutaneous tissue disorders (5588 UR, 11.73%) and circulatory system and blood disorders (5299 UR, 11.13%). Most uses are addressing mild and chronic illnesses, which agrees with the most widespread idea on the main focuses of pharmaceutical ethnobotany and phytotherapy in general [57], but in some cases, they are also pointing to acute and more severe health troubles, like cardiovascular and pulmonary ones, and even cancer.

### 2.6. Relationship between Families and Uses

One of the most important aims of this work is to study the possible relationships between plant families and categories of medicinal uses, i.e., troubles or systems addressed. We analysed the correspondences between families and health diseases, and will now comment on the most relevant findings.

**Figure 1:**
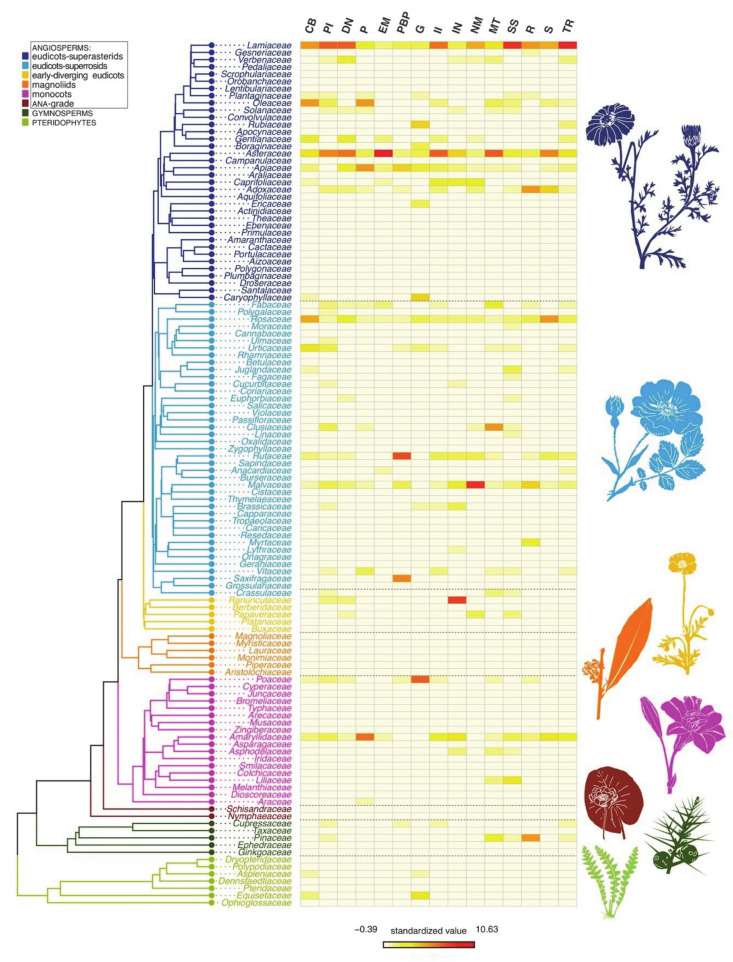
Heatmap depicting the distribution of use records among plant families and the addressed troubles/systems. Abbreviations, as quoted in the figure, are as follows. CB, circulatory system and blood disorders; PI, pain and inflammations; DN, digestives system and nutritional disorders; P, poisoning; EM, endocrine system and metabolic disorders; PBP, pregnancy, birth and puerperal disorders; G, genitourinary system disorders; II, infections and infestations; IN, immune system disorders and neoplasia; NM, nervous system and mental disorders; MT, musculoskeletal system disorders and traumas; SS, skin and subcutaneous tissue disorders; R, respiratory system disorders; S, sensory system disorders; TR, tonic and restorative.

A general consideration of the relationship between families and UR in a phylogenetic frame (Figure 1) shows, within the angiosperms, that the superasterids clearly host the largest number of uses, as well as the largest number of families with an important number of UR, in comparison with the remaining large groups. In each of these, only one or a few families play a protagonist role, such as Malvaceae, Rosaceae and Rutaceae in the superrosids, Ranunculaceae in the eudicots, and Amaryllidaceae and Poaceae in the monocots. Magnoliids and basal angiosperms are not of much significance in terms of UR. It is worth mentioning that, in the asterids, most UR are concentred in the most evolved clade, formed by the campanulids plus the lamiids. As for the gymnosperms, the Pinaceae accumulate most UR.

**Figure 2:**
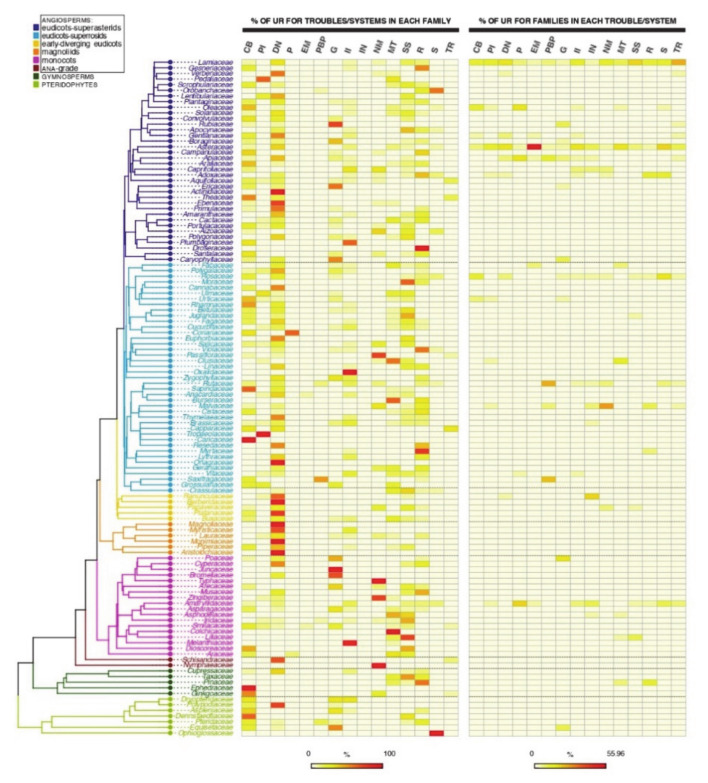
Heatmaps depicting percentages of use reports related to the different troubles/systems within each family, and percentages of use reports of families for each trouble/system. Abbreviations, as quoted in the figure, are as follows. CB, circulatory system and blood disorders; PI, pain and inflammations; DN, digestives system and nutritional disorders; P, poisoning; EM, endocrine system and metabolic disorders; PBP, pregnancy, birth and puerperal disorders; G, genitourinary system disorders; II, infections and infestations; IN, immune system disorders and neoplasia; NM, nervous system and mental disorders; MT, musculoskeletal system disorders and traumas; SS, skin and subcutaneous tissue disorders; R, respiratory system disorders; S, sensory system disorders; TR, tonic and restorative.

The analysis of the percentages of UR related to the different troubles/systems within each family (Figure 2) denotes that the highest rates are rather widespread at the phylogenetic scale. In any case, it clearly appears that a large number of families have exhibit digestive and nutritional problems as the most treated ones (mean percentage: 24.56), which is in agreement with the above-mentioned idea that ethnobotany and phytotherapy importantly address mild, daily health constraints. Nevertheless, in the vast majority of families, there are also strong rates of uses focused on circulatory and blood, and respiratory ailments (mean percentages: 14.30 and 11.03, respectively), most of which are not so mild. Finally, some disorders are very scarcely addressed in the pharmaceutical ethnoflora under consideration, such as those linked to endocrine and metabolic, and immune systems, as well as to neoplasia (mean percentages: 0.35 and 0.16, respectively).

If we analyse the percentage of use reports of families for each disorder (Figure 2), 10 out of the 15 trouble/system categories established are dominated by the two families with most UR in general, Lamiaceae (six categories: Circulatory system and blood disorders; pain and inflammation; digestive system and nutritional disorders; skin and subcutaneous tissue disorders; respiratory system disorders; tonic and restorative) and Asteraceae (four categories: Endocrine system and metabolic disorders; infections and infestations; musculoskeletal system disorders and traumas; sensory system disorders). The well-known important presence and diversity of essential oil compounds in the Lamiaceae [58] account—together with the size of the family, as already commented—for its relevance in many medicinal fields related to antiseptic properties, which could explain the prevalence in digestive, dermic and respiratory disorders. Similarly, the abundance, among other compounds, of terpene compounds (including sesquiterpene lactones) in the Asteraceae [59] is logically at the basis of their uses for different ailments, again considering the size and diversity within the family.

Concerning Asteraceae, we want to underline two troubles in particular. First, the uses for the musculoskeletal system disorders and traumas, explained by *Arnica montana* L. (335 UR, 52.59%) and other species of this family (*Arctium minus* (Hill) Bernh., *Doronicum grandiflorum* Lam., all of the *Inula* genus, *Jasonia saxatilis* (Lam.) Guss., *Pallenis spinosa* (L.) Cass. and *Pulicaria dysenterica* (L.) Gaertn.) referred to with the popular name “*àrnica*” (189 UR, 29.67%). In total, the ethnotaxon constituted by *Arnica montana* and the aforementioned related taxa accumulates 82.26% of the Asteraceae UR employed for musculoskeletal system disorders and traumas, specially bruises. This medicinal plant complex has been well studied in the Iberian Peninsula and Balearic Islands from a botanical and ethnopharmacological point of view [60]. Secondly, the uses for the endocrine system and metabolic disorders are due to hypoglycaemic activity (259 UR, 98.48%) of several species of the genus *Centaurea*, representing, with 180 UR, the 68.44% of this property, abundantly registered in this genus [61].

In the other five categories, the prominence of a family in the treatment of a problem is basically due to one or a very few taxa. Amaryllidaceae are the most quoted in fighting against poisoning cases (21.33%), with all the reports concentrated on a few species of the genus *Allium*, which has been reported with this function, and is, for instance, used worldwide against snakebites [62,63]. The dominance of Rutaceae in the pregnancy/childbirth/puerperal treatment is basically explained (27.44%) by the genus *Ruta*, with three species. This family is closely followed by the Saxifragaceae (22.56%), because of a few species of *Saxifraga*. Irrespective of the fact of containing plants used for food purposes in ethnobotany, both genera mentioned are among those most famous abortifacients recorded in folk medicine [64,65], this proving their relationship with the life period concerned (e.g., labour inducing, post-labour coadjuvant, dangerous in pregnancy). 

Poaceae are the most relevant regarding the genitourinary system (17.66%), mostly by *Zea mays* L., followed at a considerable distance by *Cynodon dactylon* (L.) Pers., both (especially the first one) are much reputed as diuretic [66]. Ranunculaceae are the top family in addressing neoplasia (21.51%), because of *Ranunculus parnassifolius* L., a high mountain plant much appreciated popularly for this purpose in a Pyrenean region [45,67]. Finally, Malvaceae leads the ranking in troubles related to the nervous system and mental disorders (29.58%). The success in this use is basically explained by the genus *Tilia* (733 UR, 97.34%)—recently incorporated into the Malvaceae, where Tiliaceae have been merged—very popular and largely studied as hypnotic, sedative and tranquilizer [68,69].

### 2.7. Phylogenetic Distribution of Families with Medicinal Use

To investigate the degree of phylogenetic clustering of families for each trouble or system, and to detect hot nodes for further studies, we mapped the reported medicinal uses grouped in the 15 troubles or systems addressed on the phylogeny of the families. 

A robust hot node appears in three medicinal groups: immune system disorders and neoplasia; pain and inflammation; and pregnancy, birth and puerperal disorders. The hot node is constituted by the Iridaceae and three families classically included in the Liliaceae l.s., Amaryllidaceae, Asparagaceae, and Asphodelaceae. The last three families also constitute a hot node clade for tonic and restorative. In addition, only Amaryllidaceae and Asparagaceae are hot nodes for the endocrine system and metabolic disorders. 

Tonic and restorative activities also have another robust hot node, constituted by Betulaceae, Juglandaceae and Fagaceae. For the endocrine system and metabolic disorders, the clade of Apiaceae and Araliacaeae was detected as relevant.

Finally, two robust hot nodes appear for poisoning, on the one hand, Cucurbitaceae and Coriariaceae, and on the other hand Malvaceae, Cistaceae and Thymelaeaceae. 

### 2.8. Diversity Indices 

The results of the Shannon and Margalef indices for each family are shown in Supplementary Materials. Although the values of the two indices are zero for several families and some of them present low values, such as the Vitaceae (H = 0.01 and k = 0.11), other families show a moderate diversity. The family Asteraceae is the one that presents the highest diversity according to the Shannon index (H = 1.45), while, according to the Margalef index, the Campanulaceae is the family with the greatest diversity (k = 0.70). These differences are due to the fact that the Margalef index is higher when the number of taxa and the number of use reports are equal or close within a family, while if there are many use reports for a few taxa, the diversity decreases. Despite showing a different sensitivity to the variation in the number of taxa and use reports, both indices are well correlated, (r = 0.841, *p* < 0.001 for a whole dataset). For this reason, we believe the two indices are robust and can be used to measure ethnobotanical diversity, even taking into account their limitations. 

### 2.9. Needs for Further Research

This study draws our attention to the relevance of the family taxonomic level in ethnobotany. A few points in which further research is needed have arisen from our analyses. 

The taxonomy of specific and infraspecific taxa in ethnobotanical works is usually given, as is logical, according to local floras but, as from the consolidation of the APG family updates, almost all papers use its system for families, which is not coincidental to those used in floras before APG rearrangements. This creates a difficulty in the comparison of data related to families in ethnobotanical research, either in one area or in different ones: the number of families and, more importantly, their rates of presence in each ethnoflora vary when applying the last classical [70] or the APG systems. An international effort should be carried out to implement the APG family system in the ethnobotanical databases in order to facilitate suitable meta-analytic work. Although new prospects will always be positive for a bigger and better knowledge of plant medicinal uses, at present a considerable amount of ethnobotanical information is already accumulated in many parts of the world, so that this is the appropriate moment to undertake comparative analyses between close and not so close ethnofloras, following the initiative, here seconded, pioneered by of Moerman [20] and followed by Weckerle et al. [22] or Dal Cero et al. [23], for which adopting the APG familiar treatment is important.

Given their relevance in most ethnofloristic surveys, the role of commercially-acquired plants in the ethnoflora, and the comparison of their uses with those of autochthonous or allochthonous plants currently present in the flora —and, then, the comparison between these two categories—is a subject that should be addressed in detail in the different major geographical areas of the world. At the family level, as treated in this work, 15 families not present in the local flora (apart from some others present hosting non-native taxa) have been recorded in a relatively small territory. This is a consequence of cultural exchanges through time, recently accelerated by the globalisation process. 

Finally, a relevant aspect is a relationship between ethnobotany and phytochemistry (the latter leading to pharmacology and linked to phylogeny). Although there is not an obvious and unidirectional relationship, and little is known about phytochemical composition as compared with evolutionary aspects [71], ethnobotanical knowledge has systematic and evolutionary significance [16,17,71], and thus can help in progressing in the necessary multidisciplinary approach to ethnopharmacology and ethnomedicine. Projecting data such as those treated here in a phylogenetic framework has allowed us to detect hot nodes, richer in families useful in pharmaceutical ethnobotany. Further deeper combination of ethnobotanical and phylogenetic information within one family or a group of a few related families could lead to detecting and predicting taxa useful for particular troubles. Several ethnobotanical works include the phytochemical and/or pharmacological validation of the folk uses reported or discuss ethnobotanical knowledge in the light of chemical plant composition [13,72,73,74,75], and this will probably be more common in the near future. Family level is particularly adequate to be addressed for establishing relationships between chemical composition, phylogenetic aspects and ethnobotanical knowledge, the three fields confronted either two by two or altogether.

## 3. Material and Methods

### 3.1. Data Sources and Field Work Methods

**Figure 3:**
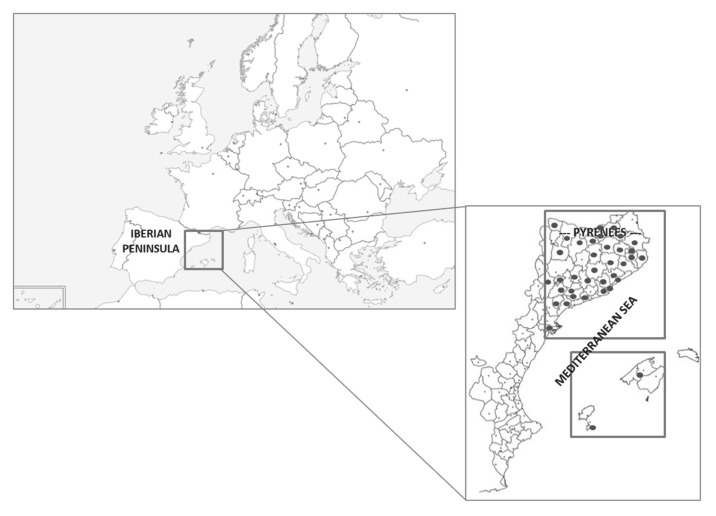
Map of the territories studied within Europe and the Catalan linguistic area. Dots indicate the areas with prospections analysed in the present paper.

In this study, we used data on plants and their folk medicinal uses obtained from 44 ethnobotanical research prospects (Supplementary Material S1) carried out in the Catalan linguistic domain (Figure 3). We utilized the use report (hereinafter, UR), i.e., the report of the use of one taxon by an informant, as the unit of measurement [76]. Veterinary uses are excluded, and human medicinal uses are classified in 15 troubles or systems categories, according to Cook [77] with minor modifications. We grouped some categories by affinity in order to achieve more robustness in the analyses, as some of them had very few reports. The names of the troubles or system categories have remained as in Cook’s classification (Table 5), and we just added a new category: tonic and restorative.

The fieldwork method used in these researches was the semistructured interview [9,78], always taking into account the code of ethics of the International Society of Ethnobiology [79], complemented by the collection of plant specimens to be identified and deposited in public herbaria. All interviews were digitalised, transcribed and introduced into our database that contains all ethnobotanical data (on medicinal, food and other uses) collected.

### 3.2. Data Analysis

The simple quantitative analyses of descriptive statistics for categories (species, families, and medicinal uses) and the construction of families and disorders matrix (Supplementary Material S2) were carried out with Excel software (Microsoft Office, 2010). Results were summarized as heatmaps using the R Phytools package ([80]; R version 3.6.0, [81]) and the family-level phylogeny of land plants from Zanne et al. [82], pruned to our taxonomic dataset. We tested whether each node in the phylogeny was significantly more represented by a family in a given category of trouble/system than would be expected by chance alone (i.e., hot nodes; [83]), using the nodesig test originally implemented in the PHYLOCOM software [84] and adapted for R by Abellán et al. [85]. 

With the aim of assessing the general state of pharmaceutical ethnobotanical knowledge in the studied area, we calculated the ethnobotanicity index (EI; [31]), which is the quotient between the number of plants used and the total number of plants that constitute the flora of the territory, expressed as a percentage. For this purpose, only the plants present in the Catalan linguistic area’s flora [25] were considered, and 5,500 taxa at specific and infraspecific level (Sáez, 2019, pers. comm.) were adopted. Furthermore, we adapted this index to calculate it for families as well.

### 3.3. Bayesian Method to Evaluate over- and Underused Taxonomic Groups

To evaluate over- and underused flora for the Catalan linguistic domain, 794 medicinal plant taxa at specific and infraspecific levels with uses reported and included of the flora of the studied area [25], belonging to 103 families, were recovered from the ethnobotanical database. The total flora for the same families of the studied area is 4,647 taxa [25]. These families were assigned to eight taxonomic groups: ANA-grade, early-diverging eudicots, eudicots-superasterids, eudicots-superrosids, gymnosperms, magnoliids, monocots and pteridophytes, following Chase et al. [48]. 

In this scenario our null hypothesis (*H*_0_) is the following: For a taxomic group (*j*), the proportion of medicinal taxa (*θj*) is equal to the overall proportion (*θ*), where *θj* is a random variable uniformily distributed between 0 and 1 (prior probability). The posterior probability can be estimated, its distribution will be conditioned by the observed data (see [22] for more details). Probability distribution differences from the common proportion are assessed by all families. 

Calculations of the intervals of the most probable values of *θ* and *θ*j were carried out by the function which returns the inverse of density of beta probability (INV.BETA.N) implemented in Excel software (Microsoft Excel 2011).

### 3.4. Diversity Indices

In order to quantify the diversity of taxa within the families referred by the informants, we calculated two diversity indices (Supplementary Material S2). The first one, the Shannon diversity index [86] from the theory of communication and largely used in ecology, has also been calculated in some previous ethnobotanical studies [87,88]. This index, calculated according to the formula H_fam_ = −Σ*p*_tax_log_2_ *p*_tax_, where *p*_tax_, represents the citation frequency of each taxon and assesses the ethnobotanical taxa diversity within each family, i.e., the family richness from the ethnobotanical point of view. The second index, k = log S/log N where S is the number of species and N the number of individuals, proposed by Margalef [89] was adapted and used for the first time in an ethnobotanical study to calculate the diversity within the botanical families following the formula: k = log T/log UR, where T represents the number of taxa. Pearson’s coefficient correlation (r) was calculated between these two datasets. 

## 4. Conclusions

The medicinal ethnoflora of the Catalan-speaking territories includes 894 taxa belonging to 120 botanical families of vascular plants. The ethnobotanicity index (EI) is 14.44% and the familial EI is 68.21% for the studied area. This parameter allows us to compare the present data with other ethnofloras. 

The most common families in the Mediterranean area, such as Lamiaceae (14.40%), Asteraceae (11.90%) or Rosaceae (5.57%) are among the most cited families which represent 57.34% of total use reports. Fagaceae, Rutaceae and Cannabaceae are the three most overused families and Cyperaceae, Plumbaginaceae and Poaceae the most underused. 

The digestive and nutritional system disorders (11,754 UR, 24.68%), the respiratory system disorders (6418 UR, 13.47%), skin or subcutaneous tissue disorders (5588 UR, 11.73%) and circulatory system and blood disorders (5299 UR, 11.13%) representing 61.01% of all use reports are the most cited troubles. 

To investigate the degree of phylogenetic clustering of families for each trouble or system and detect hot nodes for further studies we mapped the reported medicinal uses grouped in the 15 troubles or systems addressed on the phylogeny of the families. 

In the phylogenetic reconstruction, a robust hot node appears in three medicinal groups: immune system disorders and neoplasia; pain and inflammation; and pregnancy, birth and puerperal disorders constituted by the Iridaceae, Amaryllidaceae, Asparagaceae, and Asphodelaceae. The last three families also constitute a hot node clade for tonic and restorative. Tonic and restorative activities also have another robust hot node, constituted by Betulaceae, Juglandaceae and Fagaceae. For the endocrine system and metabolic disorders, the clade of Apiaceae and Araliacaeae was detected as relevant. Two robust hot nodes appear for poisoning, on the one hand Cucurbitaceae and Coriariaceae, and on the other hand Malvaceae, Cistaceae and Thymelaeaceae. These results centred on the familial level are appropriate when establishing relationships between chemical composition, phylogenetic aspects and ethnobotanical knowledge.

## Figures and Tables

**Table 1:** Top ten families by citations, with number of use reports (UR), UR percentage and number of taxa with uses present in the flora of the territories studied.

Family	UR	UR (%)	Number of Taxa with Medicinal Uses
Lamiaceae	7336	15.40	75
Asteraceae	5667	11.90	116
Rosaceae	2652	5.57	57
Malvaceae	2298	4.82	13
Adoxaceae	1971	4.14	3
Apiaceae	1661	3.49	26
Amaryllidaceae	1544	3.24	8
Oleaceae	1468	3.08	9
Pinaceae	1383	2.90	10
Rutaceae	1329	2.79	12

**Table 2:** Over- and underused high taxonomic groups in the Catalan-speaking territories.

Taxonomic Group (*J*)	*n_j_*	*x_j_*	Inf.	*Θj*	Sup.
Gymnosperms *	34	19	0.394	0.56	0.712
Eudicots-superrosids	1401	273	0.175	0.19	0.216
Pteridophytes	69	18	0.172	0.26	0.376
Eudicots-superasterids	2178	395	0.166	0.18	0.198
ANA-grade	1	1	0.158	1.00	0.987
Magnoliids	7	3	0.157	0.43	0.755
Early-diverging eudicots	175	22	0.085	0.13	0.183
Monocots *	782	63	0.064	0.08	0.102
Total/common	4647	794	0.160	0.17	0.182

*n_j_*: number of plant taxa in the overall flora; *x_j_*: number of medicinal plant taxa; *θj*: proportion of medicinal species; Inf. and Sup.: The 95% posterior credible interval of *θj.* * Taxonomic groups differing from *H*_0_.

**Table 3:** Overused plant families in the studied area.

Family (*J*)	*n_j_*	*x_j_*	Inf.	*θj*	Sup.	Margin
Fagaceae	15	11	0.476	0.73	0.890	0.294
Rutaceae	15	11	0.476	0.73	0.890	0.294
Cannabaceae	3	3	0.398	1.00	0.994	0.216
Pinaceae	15	9	0.354	0.60	0.802	0.172
Cucurbitaceae	13	8	0.351	0.62	0.823	0.169
Equisetaceae	8	5	0.299	0.63	0.863	0.117
Cupresaceae	15	8	0.299	0.53	0.753	0.117
Oleaceae	15	8	0.299	0.53	0.753	0.117
Buxaceae	2	2	0.292	1.00	0.992	0.110
Apocynaceae	13	7	0.289	0.54	0.770	0.107
Rhamnaceae	13	7	0.289	0.54	0.770	0.107
Myrtaceae	4	3	0.284	0.75	0.947	0.102
Lamiaceae	207	67	0.264	0.32	0.390	0.082
Asphodelaceae	12	6	0.251	0.50	0.749	0.069
Urticaceae	10	5	0.234	0.50	0.766	0.052
Crassulaceae	38	14	0.234	0.37	0.528	0.052
Ericaceae	22	9	0.232	0.41	0.615	0.050
Rosaceae	190	54	0.225	0.28	0.352	0.043
Solanaceae	37	13	0.218	0.35	0.514	0.036
Araceae	11	5	0.211	0.45	0.723	0.029
Polypodiaceae	3	2	0.194	0.67	0.932	0.012
Adoxaceae	6	3	0.184	0.50	0.816	0.002

*n_j_*: number of plant taxa in the overall flora; *x_j_*: number of medicinal plant taxa; *θj*: proportion of medicinal species; Inf. and Sup.: The 95% posterior credible interval of *θj*.

**Table 4:** Underused plant families in the studied area.

Family (*J*)	*n_j_*	*x_j_*	Inf.	*Θj*	Sup.	Margin
Fabaceae	383	45	0.089	0.12	0.154	0.006
Ranunculaceae	126	9	0.038	0.07	0.130	0.030
Brassicaceae	256	22	0.058	0.09	0.127	0.033
Orobanchaceae	78	4	0.021	0.05	0.125	0.035
Caryophyllaceae	233	18	0.050	0.08	0.119	0.041
Juncaceae	45	1	0.005	0.02	0.115	0.045
Poaceae	417	22	0.035	0.05	0.079	0.081
Plumbaginaceae	80	1	0.003	0.01	0.067	0.093
Cyperaceae	131	2	0.005	0.02	0.054	0.106

*n_j_*: number of plant taxa in the overall flora; *x_j_*: number of medicinal plant taxa; *θj*: proportion of medicinal species; Inf. and Sup.: The 95% posterior credible interval of *θj*.

**Table 5:** Medicinal uses grouped in troubles or systems addressed with number and percentage of use reports (UR).

Troubles or Systems Addressed (Code)	UR	UR (%)
Digestive system and nutritional disorders (DN)	11,754	24.68
Respiratory system disorders (R)	6418	13.47
Skin and subcutaneous tissue disorders (SS)	5588	11.73
Circulatory system and blood disorders (CB)	5299	11.13
Infections and infestations (II)	3831	8.04
Genitourinary system disorders (G)	3618	7.60
Musculoskeletal system disorders and traumas (MT)	3493	7.33
Nervous system and mental disorders (NM)	2546	5.35
Sensory system disorders (S)	1975	4.15
Pain and inflammations (PI)	1224	2.57
Pregnancy, birth and puerperal disorders (PBP)	554	1.16
Endocrine system and metabolic disorders (EM)	470	0.99
Tonic and restorative (TR)	406	0.85
Poisoning (P)	361	0.76
Immune system disorders and neoplasia (IN)	93	0.20

## Data Availability

The data presented in this study are available in Supplementary Material S2, and further information could bo obtained on request from the corresponding authors.

## Supplementary Materials

The following are available online at https://www.mdpi.com/2223-7747/10/1/163/s1, **S1**. References used to elaborate the data matrix analysed; **S2**. Families and disorders matrix, and diversity indices.

## References

[1] Harshberger J.W. (1896). Purposes of ethno-botany. Bot. Gaz..

[2] de Rochebrune A.T. (1879). Recherches d’ethnographie botanique sur la flore des sepultures péruviennes d’Ancon. Actes Soc. Linn. Bordx..

[3] Pardo-de-Santayana M., Macía M.J. (2015). The benefits of traditional knowledge. Nature.

[4] Gras A., Garnatje T., Bonet M., Carrió E., Mayans M., Parada M., Rigat M., Vallès J. (2016). Beyond food and medicine, but necessary for life, too. Other folk plant uses in several territories of Catalonia and the Balearic Islands. J. Ethnobiol. Ethnomed..

[5] Tringali C. (2012). Bioactive Compounds From Natural Sources: Natural Products as Lead Compounds in Drug Discovery.

[6] Tu Y. (2016). Artemisinin—A gift from traditional Chinese medicine to the World (Nobel lecture). Angew. Chem. Int. Ed..

[7] Pardo-de-Santayana M., Pieroni A., Puri R.K. (2013). Ethnobotany in the New Europe: People, Health and Wild Plant Resources. Environmental Anthropology and Ethnobiology.

[8] Pardo-de-Santayana M., Quave C.L., Söukland R., Pieroni A., Heinrich M., Jäger A.K. (2015). Medical ethnobotany and ethnopharmacology of Europe. Ethnopharmacology.

[9] Vallès J. (2019). Etnobotànica: Persones, Plantes, Cultura i Benestar. Aspectes Generals, i Situació i Perspectives al Països Catalans.

[10] Gras A., Parada M., Vallès J., Garnatje T. (2020). Catalan ethnoflora: A meta-analytic approach to life forms and geographic territories. J. Ethnobiol. Ethnomed..

[11] Gras A., Serrasolses G., Vallès J., Garnatje T. (2019). Traditional knowledge in semi-rural close to industrial areas: Ethnobotanical studies in western Gironès (Catalonia, Iberian Peninsula). J. Ethnobiol. Ethnomed..

[12] Gras A., Vallès J., Garnatje T. (2020). Filling the gaps: Ethnobotanical study of the Garrigues district, an arid zone in Catalonia (NE Iberian Peninsula). J. Ethnobiol. Ethnomed..

[13] Rigat M., Vallès J., D’Ambrosio U., Gras A., Iglésias J., Garnatje T. (2015). Plants with topical uses in the Ripollès district (Pyrenees, Catalonia, Iberian Peninsula). ethnobotanical survey and pharmacological validation in the literature. J. Ethnopharmacol..

[14] Rønsted N., Symonds M.R.E., Birkholm T., Christensen S.B., Meerow A.W., Molander M., Mølgaard P., Petersen G., Rasmussen N., Staden J. (2012). Can phylogeny predict chemical diversity and potential medicinal activity of plants? A case study of amaryllidaceae. BMC Evol. Biol..

[15] Saslis-Lagoudakis C.H., Savolainen V., Williamson E.M., Forest F., Wagstaff S.J., Baral S.R., Watson M.F., Pendry C.A., Hawkins J.A. (2012). Phylogenies reveal predictive power of traditional medicine in bioprospecting. Proc. Natl. Acad. Sci. USA.

[16] Garnatje T., Peñuelas J., Vallès J. (2017). Ethnobotany, phylogeny, and ‘omics’ for human health and food security. Trends Plant Sci..

[17] Garnatje T., Peñuelas J., Vallès J. (2017). Reaffirming ’Ethnobotanical Convergence’. Trends Plant Sci..

[18] Alrashedy N.A., Molina J. (2016). The ethnobotany of psychoactive plant use: A phylogenetic perspective. PeerJ..

[19] Pellicer J., Saslis-Lagoudakis C.H., Carrió E., Ernst M., Garnatje T., Grace O.M., Gras A., Mumbrú M., Vallès J., Vitales D. (2018). A phylogenetic roadmap to antimalarial Artemisia species. J. Ethnopharmacol..

[20] Moerman D.E. (1996). An analysis of the food plants and drug plants of native North America. J. Ethnopharmacol..

[21] Moerman D.E., Pemberton R.W., Kiefer D., Berlin B. (1999). A comparative analysis of five medicinal floras. J. Ethnobiol..

[22] Weckerle C.S., Cabras S., Castellanos M.E., Leonti M. (2011). Quantitative methods in ethnobotany and ethnopharmacology: Considering the overall flora—Hypothesis testing for over-and underused plant families with the Bayesian approach. J. Ethnopharmacol..

[23] Dal Cero M., Saller R., Weckerle C.S. (2014). The use of the local flora in Switzerland: A comparison of past and recent medicinal plant knowledge. J. Ethnopharmacol..

[24] Bennett B.C., Husby C.E. (2008). Patterns of medicinal plant use: An examination of the Ecuadorian Shuar medicinal flora using contingency table and binomial analysis. J. Ethnopharmacol..

[25] Bolòs O., de Vigo J., Masalles R.M., Ninot J.M. (2005). Flora Manual dels Països Catalans.

[26] Novais M.H., Santos I., Mendes S., Pinto-Gomes C. (2004). Studies on Pharmaceutical Ethnobotany in Arrábida Natural Park (Portugal). J. Ethnopharmacol..

[27] Tuttolomondo T., Licata M., Leto C., Savo V., Bonsangue G., Gargano M.L., Venturella G., La Bella S. (2014). Ethnobotanical investigation on wild medicinal plants in the Monti Sicani Regional Park (Sicily, Italy). J. Ethnopharmacol..

[28] Menendez-Baceta G., Aceituno-Mata L., Molina M., Reyes-García V., Tardío J., Pardo-de-Santayana M. (2014). Medicinal plants traditionally used in the northwest of the Basque Country (Biscay and Alava), Iberian Peninsula. J. Ethnopharmacol..

[29] Camejo-Rodrigues J.S., Ascensão L., Bonet M.À., Vallès J. (2003). An Ethnobotanical Study of Medicinal and Aromatic Plants in the Natural Park of “Serra de São Mamede” (Portugal). J. Ethnopharmacol..

[30] Mary D.A., Franco F.M., Babu V. (2011). Assessing the Contribution of Local and Traded Biodiversity in Community Health Care: A case study from Keelakodankulam village, South India. Ethnobot. Res. Appl..

[31] Portères R. (1970). Cours d’Ethno-Botanique et Ethno-Zoologie (1969–1970), Ethno-botanique générale.

[32] Funk V.A., Sussana A., Stuessy T.F., Bayer R.J. (2009). Systematics, Evolution, and Biogeography of Compositae.

[33] Kadereit J.W. (2012). Flowering Plants·Dicotyledons: Lamiales (Except Acanthaceae Including Avicenniaceae).

[34] Hummer K.E., Janick J., Folta L.M., Gardiner S.E. (2009). Rosaceae: Taxonomy, economic importance, genomics. Genetics and Genomics of Rosaceae.

[35] Pieroni A., Quave C., Nebel S., Heinrich M. (2002). Ethnopharmacy of the ethnic Albanians (Arbëreshë) of northern Basilicata, Italy. Fitoterapia.

[36] Pieroni A., Quave C.L., Santoro R.F. (2004). Folk pharmaceutical knowledge in the territory of the Dolomiti Lucane, inland southern Italy. J. Ethnopharmacol..

[37] Scherrer A.M., Motti R., Weckerle C.S. (2005). Traditional plant use in the areas of Monte Vesole and Ascea, Cilento National Park (Campania Southern Italy). J. Ethnopharmacol..

[38] Akerreta S., Cavero R.Y., Calvo M.I. (2007). First comprehensive contribution to medical ethnobotany of Western Pyrenees. J. Ethnobiol. Ethnomed..

[39] Maxia A., Lancioni M.C., Balia A.N., Alborghetti R., Pieroni A., Loi M.C. (2007). Medical ethnobotany of the Tabarkins, a Northern Italian (Ligurian) minority in south-western Sardinia. Gen. Res. Crop Evol..

[40] Guarrera P.M., Forti G., Marignoli S. (2005). Ethnobotanical and ethnomedicinal uses of plants in the district of Acquapendente (Latium Central Italy). J. Ethnopharmacol..

[41] Guarrera P.M., Lucchese F., Medori S. (2008). Ethnophytotherapeutical research in the high Molise region (Central-Southern Italy). J. Ethnobiol. Ethnomed..

[42] González-Tejero M.R., Casares-Porcel M., Sánchez-Rojas C.P., Ramiro-Gutiérrez J.M., Molero-Mesa J., Pieroni A., Giusti M.E., Censorii E., de Pasquale C., Della A. (2008). Medicinal plants in the Mediterranean area: Synthesis of the results of the project Rubia. J. Ethnopharmacol..

[43] Carrió E., Vallès J. (2012). Ethnobotany of medicinal plants used in Eastern Mallorca (Balearic Islands, Mediterranean Sea). J. Ethnopharmacol..

[44] Menale B., De Castro O., Cascone C., Muoio R. (2016). Ethnobotanical investigation on medicinal plants in the Vesuvio National Park (Campania, Southern Italy). J. Ethnopharmacol..

[45] Rigat M., Gras A., Vallès J., Garnatje T. (2017). Estudis etnobotànics a la comarca del Ripollès (Pirineu, Catalunya, península Ibèrica). Collect. Bot..

[46] Johns T., Kokwaro J.O., Kimanani E.K. (1990). Herbal remedies of the Luo of Siaya District, Kenya: Establishing quantitative criteria for consensus. Econ. Bot..

[47] Bonet M.À., Parada M., Selga A., Vallès J. (1999). Studies on pharmaceutical ethnobotany in the regions of L’Alt Emporda and Les Guilleries (Catalonia, Iberian Peninsula). J. Ethnopharmacol..

[48] Chase M.W., Christenhusz M.J., Fay M.F., Byng J.W., Judd W.S., Soltis D.E., Mabberley D.J., Sennikov A.N., Soltis P.S., Stevens P.F. (2016). An update of the Angiosperm Phylogeny Group classification for the orders and families of flowering plants: APG IV. Bot. J. Linn. Soc..

[49] Christenhusz M.J.M., Fay M.F., Chase M.W. (2017). Plants of the World: An Illustrated Encyclopedia of Vascular Plants.

[50] Vallès J., Bonet M.À., Agelet A. (2004). Ethnobotany of *Sambucus nigra* L.: The integral exploitation of a natural resource in mountain regions of Catalonia (Iberian Peninsula). Econ Bot..

[51] Vallès J., Bonet M.À., Garnatje T., Muntané J., Parada M., Rigat M., Peter K.V. (2010). Sambucus nigra L. In Catalonia (Iberian Peninsula): Popular knowledge and holistic exploitation of an underutilised natural resource. Underutilized and Underexploited Horticultural Crops.

[52] Moerman D.E., Etkin N.L. (1994). Native American food and drug plants. Eating on the Wild Side: The Pharmacologic, Ecologic, and Social Implications of Using Noncultigens.

[53] Stepp J.R., Moerman D.E. (2001). The importance of weeds in ethnopharmacology. J. Ethnopharmacol..

[54] Leonti M., Casu L., Sanna F., Bonsignore L. (2009). A comparison of medicinal plant use in Sardinia and Sicily-De *Materia Medica* revisited?. J. Ethnopharmacol..

[55] Masclans F. (1949). Aspecte general de la vegetació en la conca del Gaià. Inst. Cat. Hist. Nat..

[56] Brunés M. (2017). La Globalització en el Saber Popular. Plantes Medicinals de fora en la Tradició d’aquí. Bachelor Thesis.

[57] Barnes J. (2003). Quality, efficacy and safety of complementary medicines: Fashions, facts and the future. Part I. Regulation and quality. Br. J. Clin. Pharmacol..

[58] Lawrence B.M., Harley R.M., Reynolds T. (1992). Chemical components of Labiatae oils and their exploitation. Advances in Labiate Science.

[59] Calabria L.M., Emerenciano V.P., Scotti M.T., Mabry T.J., Funk V.A., Sussana A., Stuessy T.F., Bayer R.J. (2009). Secondary chemistry of Compositae. Systematics, Evolution, and Biogeography of Compositae.

[60] Obón C., Rivera D., Verde A., Fajardo J., Valdés A., Alcaraz F., Carvalho A.M. (2012). Árnica: A multivariate analysis of the botany and ethnopharmacology of a medicinal plant complex in the Iberian Peninsula and the Balearic Islands. J. Ethnopharmacol..

[61] Subramoniam A. (2016). Plants with Anti-Diabetes Mellitus Properties.

[62] Houghton P.J., Osibogun I.M. (1993). Flowering plants used against snakebites. J. Ethnopharmacol..

[63] Upasani. S.V., Beldar V.G., Tatiya A.U., Upasani M.S., Surana S.J., Patil D.S. (2017). Ethnomedicinal plants used for snakebite in India: A brief overview. Integr. Med. Res..

[64] Bonet M.À., Vallès J. (2003). Pharmaceutical ethnobotany in the Montseny biosphere reserve (Catalonia, Iberian Peninsula). General results and new or rarely reported medicinal plants. J. Pharm. Pharmacol..

[65] Freitas T.G., de Augusto P.M., Montanari T. (2005). Effect of *Ruta graveolens* L. on pregnant mice. Contraception..

[66] Wright C.I., Van Buren L., Kroner C.I., Konning M.M. (2007). Herbal medicines as diuretics: A review of the scientific evidence. J. Ethnopharmacol..

[67] Rigat M., Bonet M.À., Garcia S., Garnatje T., Vallès J. (2007). Studies on pharmaceutical ethnobotany in the high river Ter valley (Pyrenees, Catalonia, Iberian Peninsula). J. Ethnopharmacol..

[68] Aguirre-Hernández E., Martínez A.L., González-Trujano M.E., Moreno J., Vibrans H., Soto-Hernández M. (2007). Pharmacological evaluation of the anxiolytic and sedative effects of *Tilia americana* L. var.*mexicana* in mice. J. Ethnopharmacol..

[69] Rezaie A., Jafari B., Ahmadizadeh C., Jalizadeh Hedayati M., Ostadi Z., Ebadi A.R., Shishegar R. (2011). Study of sedative preanaesthetic and anxiolytic effects of herbal extract of *Tilia platyphyllos* Scop. In comparison with diazepam in the rat. Vet. Clin. Pathol..

[70] Cronquist A. (1981). An Integrated System of Classification of Flowering Plants.

[71] Gottlieb O.R., Borin M.R.M.B., Brito N.R.S. (2002). Integration of ethnobotany and phytochemistry: Dream or reality?. Phytochemistry.

[72] Calvo M.I., Akerreta S., Cavero R.Y. (2013). Medicinal plants used for dermatological affections in Navarra and their pharmacological validation. J. Ethnopharmacol..

[73] Cavero R.Y., Akerreta S., Calvo M.I. (2013). The pharmacological validation of medicinal plants used for digestive problems in Navarra, Spain. Eur. J. Integr. Med..

[74] Heinrich M., Booker A., Heinrich M., Jäger A.K. (2015). Can there be an ethnopharmacology of inflammation. Ethnopharmacology.

[75] Hensel A., Kisseih E., Lechtenberg M., Petereid F., Agyare C., Asase A., Heinrich M., Jäger A.K. (2015). From ethnopharmacological field study to phytochemistry and preclinical research: The example of Ghanaian medicinal plants for improved wound healing. Ethnopharmacology.

[76] Vandebroek I., Thomas E., Sanca S., Van Damme P., Van Puyelde L., De Kimpe N. (2008). Comparison of health conditions treated with traditional and biomedical health care in a Quechua community in rural Bolivia. J. Ethnobiol. Ethnomed..

[77] Cook F.E.M. (1985). Economic Botany Data Collection Standard.

[78] Pujadas J.J., Comas D., Roca J. (2004). Etnografia.

[79] International Society of Ethnobiology International Society of Ethnobiology Code of Ethics (with 2008 Additions). http://ethnobiology.net/code-of-ethics.

[80] Revell L.J. (2012). Phytools: An R package for phylogenetic comparative biology (and other things). Meth. Ecol. Evol..

[81] R Core Team (2019). R: A Language and Environment for Statistical Computing.

[82] Zanne A.E., Tank D.C., Cornwell W.K., Eastman J.M., Smith S.A., FitzJohn R.G., McGlinn D.J., O’Meara B.C., Moles A.T., Reich P.B. (2014). Three keys to the radiation of angiosperms into freezing environments. Nature.

[83] Saslis-Lagoudakis C.H., Klitgaard B.B., Forest F., Francis L., Savolainen V., Williamson E.M., Hawkins J.A. (2011). The use of phylogeny to interpret cross-cultural patterns in plant use and guide medicinal plant discovery: An example from Pterocarpus (Leguminosae). PLoS ONE.

[84] Webb C.O., Ackerly D.D., Kembel S.W. (2008). Phylocom: Software for the analysis of phylogenetic community structure and trait evolution. Bioinformatics.

[85] Abellán P., Carrete M., Anadón J.D., Cardador L., Tella J.L. (2016). Non-random patterns and temporal trends (1912–2012) in the transport, introduction and establishment of exotic birds in Spain and Portugal. Divers. Distrib..

[86] Shannon C.E. (1948). A mathematical theory of communication. Bell Syst. Techn. J..

[87] Begossi A. (1996). Use of ecological methods in ethnobotany: Diversity indices. Econ. Bot..

[88] Gras A., Parada M., Rigat M., Vallès J., Garnatje T. (2018). Folk medicinal plant mixtures: Establishing a protocol for further studies. J. Ethnopharmacol..

[89] Margalef R. (1989). On diversity and connectivity, an historical expression of ecosystems. Coenoses.

