# Supplementary material for: The Role of Botanical Families in Medicinal Ethnobotany: A Phylogenetic Perspective
Source: Plants (Basel). 2021 Jan 15;10(1):163. doi: 10.3390/plants10010163 (PMC7830233; doi:10.3390/plants10010163)
Supplement: Supplementary file 1 [file plants-10-00163-s001.zip › sup/Supplementary material 1.pdf]

Supplementary material 1

References used to elaborate the data matrix analysed.

Agelet, A. Estudis d'etnobotànica farmacèutica al Pallars. PhD thesis, Universitat de Barcelona, 1999.

Almeida, B.; Aldea, C. Estudi etnobotànic de la Vall d'Aran i recerca sobre aspectes actuals de les plantes medicinals. High school research work, Vielha, 2015.

Alòs, E. Introducció a l'etnobotànica del Delta de l'Ebre. Undergraduate course research work. Universitat de Barcelona, 2019.

Altimiras, J.; Casassas, E.; Montaña, D. Remeis populars a les Valls d'Aguilar. Edicions Salòria: la Seu d'Urgell, 2011.

Batet, D.; Cartanyà, J.; Castells, R.; Piñas, I.; Salat, X. Etnobotànica a les muntanyes de Prades. Centre d'Història Natural de la Conca de Barberà: Montblanc, 2011.

Benito, C.; Massó, S.; Merlo, R. Treball etnobotànic del Baix Penedès. Master course research work, Universitat de Barcelona, 2011.

Blanch, A. Estudi etnobotànic de la regió geogràfica del Massís de les Gavarres. Undergraduate course research work, Universitat de Barcelona, 2013.

Bonet, M.À. Estudis etnobotànics a la Vall del Tenes (Vallès Oriental). BSc thesis, Universitat de Barcelona, 1991.

Bonet, M.À. Estudi etnobotànic del Montseny. PhD thesis, Universitat de Barcelona, 2001.

Bonet, M.À.; Roldán, M.; Camprubí, J.; Vallès, J. Etnobotànica de Gallecs. Plantes i cultura popular al Baix Vallès. Centre d'Estudis Molletans: Mollet del Vallès, 2008.

Calvo, A. Treball etnobotànic: els rials d'Arenys. Undergraduate course research work, Universitat de Barcelona, 2019.

Cano, M. Estudi etnobotànic al Bages. L'ús tradicional de les plantes de la vall del Cardener. High school research work, Manresa, 2016.

Capdevila, M. Estudi etnobotànic d'Ivars d'Urgell. Undergraduate course research work, Universitat de Barcelona, 2014.

Carrió, E. Contribució a l'etnobotànica de Mallorca. La biodiversitat vegetal i la seva gestió en una illa mediterrània. PhD thesis, Universitat de Barcelona, 2013.

Cirera, E. Etnobotànica de Sant Feliu Sassera (Lluçanès). Unpublished research, Universitat de Barcelona, Institut Botànic de Barcelona.

Díaz, E. Iniciació a la recerca etnobotànica. Estudi etnobotànic del Ripollès. Undergraduate course research work, Universitat de Barcelona, 2009.

Gras, A.; Vallès, J.; Garnatje, T. Filling the gaps: ethnobotanical study of the Garrigues district, an arid zone in Catalonia (NE Iberian Peninsula). *J. Ethnobiol. Ethnomed.* **2020**, *16(1)*, 1-15.

Gras, A.; Garnatje, T.; Álvarez, C.; Herruzo, M.; Jané, N.; Vallès, J. Etnobotànica del Parc Natural de Collserola. Unpublished research, Universitat de Barcelona, Institut Botànic de Barcelona.

Gumà, I. Treball etnobotànic a l'Anoia. Undergraduate course research work, Universitat de Barcelona, 2004.

Jané, N. Treball etnobotànic a la Serra de Collserola. Undergraduate course research work, Universitat de Barcelona, 2008.

Lluís, A. Estudi etnobotànic del Molar. Undergraduate course research work, Universitat de Barcelona, 2010.

Llurba, N. Estudi etnobotànic d'Ulldemolins (el Priorat). Undergraduate course research work, Universitat de Barcelona, 2009.

Lozano, P. Treball etnobotànic de Gironella. Undergraduate course research work, Universitat de Barcelona, 2008.

Marín, J. Estudis etnobotànics al Baix Llobregat. Unpublished research, part of a PhD thesis in progress, Universitat de Barcelona.

Mayans, M. Estudi etnobotànic de Formentera. Master thesis, Universitat de Barcelona, 2013.

Mayor, L. Etnobotànica a l'Anoia. Treball de curs de Botànica econòmica, Facultat de Farmàcia, Universitat de Barcelona, 2009.

Muntané, J. Aportació al coneixement de l'etnobotànica de Cerdanya. PhD thesis, Universitat de Barcelona, 1991.

Muntané, J. Etnobotànica, etnofarmàcia i tradicions populars de la Catalunya septentrional (Capcir, Cerdanya, Conflent). PhD thesis, Universitat de Barcelona, 2005.

Nogueras, A. Estudi etnobotànic a Martorelles, Santa Maria de Martorelles i Sant Fost de Campsentelles (Vallès Oriental, Catalunya). BSc thesis, Universitat de Barcelona, 2013.

Olivieri, M.I. Estudi etnobotànic de la comarca del Segrià. Undergraduate course research work, Universitat de Barcelona, 2010.

Pagès, L. Estudi etnobotànic al municipi de Cassà de la Selva. BSc thesis, Facultat de Farmàcia, Universitat de Barcelona, 2013.

Parada, M. Estudi etnobotànic de l'Alt Empordà. PhD thesis, Universitat de Barcelona, 2008.

Plana, E. Estudi etnobotànic de la Vall d'en Bas. Undergraduate course research work, Universitat de Barcelona, 2013.

Planas, I. L'ús de les plantes al Pla de l'Estany. Master course research work, Universitat de Barcelona, 2011.

Raja, D. Estudis etnobotànics a la comarca de la Segarra. BSc thesis, Universitat de Barcelona, 1995.

Rigat, M.; Gras, A.; Vallès, J.; Garnatje, T. Estudis etnobotànics a la comarca del Ripollès (Pirineu, Catalunya, península Ibèrica). Collect. Bot. (Barcelona), **2017**, *36*, e003.

Roig, M. Estudi etnobotànic a la Selva del Camp i l'Albiol. Undergraduate course research work, Universitat de Barcelona, 2019.

Sala, E. Etnobotànica del Pla de l'Estany. Unpublished research in progress, Universitat de Barcelona, Institut Botànic de Barcelona, 2019.

Saura, S. Usos i cultura popular de les plantes a les Gavarres. Consorci de les Gavarres: Monells, 2009.

Selga, A. Estudis etnobotànics a les Guilleries. BSc thesis, Universitat de Barcelona, 1998.

Serrasolses, G. Estudi etnobotànic del Gironès occidental. Master thesis, Universitat de Barcelona, 2014.

Solé, M. Estudi etnobotànic de Valls. Undergraduate course research work, Universitat de Barcelona, 2013.

Sorribas, M. Estudi etnobotànic de Vilanova de Bellpuig. Undergraduate course research work, Universitat de Barcelona, 2011.

Usles, E. Estudi etnobotànic de l'Alt Berguedà. Undergraduate course research work, Universitat de Barcelona, 2011.
